# The impact of cardiac resynchronization therapy on diastolic parameters and mitral regurgitation: echocardiographic analysis of ultrasound-based left ventricular endocardial pacing system

**DOI:** 10.3389/fcvm.2026.1768362

**Published:** 2026-03-18

**Authors:** Ryan Wilson, Dinesh Sharma, Simon James, Shunsuke Eguchi, Yoshiyuki Orihara, Viviana Navas, Rahul Bhardwaj, Michael Pfeiffer, Spencer H. Kubo

**Affiliations:** 1 Penn State College of Medicine, Hershey PA, United States; 2 NCH Healthcare System, Naples, Florida; 3 NHS South Tees Hospital, Northallerton, United Kingdom; 4 Loma Linda University Hospital, Loma Linda CA, United States; 5 United Hospital, St. Paul MN, United States

**Keywords:** biventricular pacing, cardiac resynchronization therapy, diastolic function, echocardiography, heart failure, mitral regurgitation

## Abstract

**Background:**

The WiSE-CRT system offers an innovative approach to biventricular pacing in individuals who previously were considered too high risk or had failed traditional transvenous CRT therapy. The impact on LV volumes and systolic function have previously been published. This study evaluates the impact of the WiSE CRT system on diastolic filling parameters and mitral regurgitation.

**Methods:**

Retrospective cohort study of patients in SOLVE-CRT with device implantation, activated CRT therapy, and a 6-month follow-up TTE. Baseline and 6-month parameters of diastolic function and mitral regurgitation were evaluation. Most patients had sufficient data for analysis. A sub-group analysis examined results in ischemic and non-ischemic cardiomyopathies.

**Results:**

Individual parameters of diastolic function and mitral regurgitation trended towards improvement at 6-months. Summative classification of diastolic function and mitral regurgitation both showed statistically significant improvement at 6-months follow-up (*p* < 0.01).

**Conclusion:**

In addition to the established improvement in LV volumes and LV EF, patients with the WiSE-CRT biventricular pacing system demonstrate improvement in aggregate assessment of diastolic function and mitral regurgitation at 6-months.

## Introduction

The WiSE-CRT system (EBR Systems, Sunnyvale, CA) offers an innovative approach to biventricular pacing (BiVP). The system consists of a Battery connected to an ultrasound Transmitter that is implanted subcutaneously in the fourth to sixth intercostal space and a Receiver Electrode that is implanted endocardially in the left ventricle (LV). The Transmitter senses the right ventricle (RV) pacing spike of the co-implant device and within ∼5 ms emits an ultrasound pulse to the Electrode, which converts the ultrasound pulse into electrical energy sufficient to pace the LV, thereby resulting in BiVP (1, 2). In the initial SOLVE-CRT study, patients were included who previously had failed coronary sinus (CS) lead implantation or who were considered high risk for implantation of a cardiac resynchronization therapy (CRT) system. The findings of the index study demonstrated a high success rate of LV endocardial pacing, as well favorable clinical responses in heart failure symptoms, and significant LV remodeling with reductions in overall LV volumes and improvement in LV ejection fraction (LVEF) (2).

With the noted improvement in volumetric LV parameters, there was a question regarding the effects of BiVP with the WiSE-CRT system on other echocardiographic parameters such as diastolic filling and mitral regurgitation (MR). Previous studies evaluating changes in diastolic parameters with traditional transvenous CRT devices have been mixed in their results (3–8). Some studies have shown a positive correlation between improvement in LV remodeling post CRT implantation with a concomitant improvement in LV filling pressures and diastolic parameters (3, 7–9).

The principal objective of this research was to investigate the impact of the WiSE-CRT system on LV filling pressures, diastolic parameters, and MR.

## Methods

### Study participants

This is a single-arm, retrospective, observational study. The study population consisted of 87 patients from the initial SOLVE-CRT study who underwent device implantation, with active CRT therapy, and had both baseline and 6 month follow up echocardiograms for review. The inclusion criteria for patients in the initial study included patients who had previously failed conventional CS implantation or who were considered to have an elevated risk for implantation of a traditional transvenous CRT upgrade. The major exclusion criteria included right bundle branch block, LV end-diastolic diameter >8 cm, life expectancy < 1 year, patients with recent acute coronary syndrome, and Grade 4 MR (1, 2). The complete inclusion and exclusion criteria for patients in the SOLVE-CRT study were previously published in detail (1).

Baseline characteristics, reason for device implantation, and type of cardiomyopathy were obtained from the initial study database. Prior to implantations, all patients underwent a transthoracic echocardiogram (TTE). Follow up echocardiograms were performed 6 months after implantation of CRT device.

### Echocardiographic evaluation

Evaluation of LVEF and LV volumes, including LV End Diastolic Volume (LVEDV) and, LV End Systolic Volume (LVESV) were calculated using biplane volumetric assessment. Diastolic parameters were measured in accordance with American Society of Echocardiography guidelines. Tissue Doppler was performed at both the septal and lateral annuli to record tissue velocities. Left Atrial Volume Index (LAVI) was calculated using the area—length measurement. Grades of diastolic dysfunction (I-III) were given for patients at baseline and 6 months based on the 2016 American Society of Echocardiography diastolic guidelines (10). Given the presence of structural heart disease in all patients in this cohort, presented in Figure 8 (B) in the guideline document. Patients that were missing diagnostic information or those who based on variables presented in the algorithm had mixed information were classified as “indeterminate” diastolic grade in accordance with the guideline statement (10).

MR was evaluated primarily in a qualitative fashion. Degree of MR was classified qualitatively on a standard 0-4 scale (0 = none/trace, 1 = mild, 2 = moderate, 3 = moderate-severe, 4 = severe). Quantitative evaluation was done using vena-contracta of MR jet width. Vena contracta measurements were performed in a consistent approach from an apical 4 chamber view (11).

All echocardiographic parameters were analyzed by the echocardiographic core laboratory (Pennsylvania State University College of Medicine, Hershey, PA, USA). The core lab team was blinded to all clinical and outcomes data. Echocardiographic measurements were primarily performed using a single representative cardiac cycle. In patients with regular rhythms, one cardiac cycle was analyzed. In patients with an irregular rhythm, a representative beat with an average RR interval was selected. Average across multiple cardiac cycles was performed only in rare cases when no representative cycle could be identified. Analysis was performed offline using vendor-independent software (TomTec/Phillips).

### Study endpoints

The primary endpoints in this retrospective analysis included comparison of diastolic parameters and MR assessment from baseline to 6 months following WiSE-CRT device implantation. Secondary endpoints compared patients with ischemic cardiomyopathy vs. non-ischemic cardiomyopathy.

### Statistical analysis

Categorical data were summarized as the proportion of subjects exhibiting the endpoint of interest. Continuous endpoints were summarized as a mean, standard deviation, and sample size. To compare performance values between preimplant (baseline) and follow-up intervals, a paired Student *t*-test was used for normally distributed data, the Wilcoxon signed rank test was used for non-normally distributed data, and the Fisher's exact test was used for categorical data. A *p* value < 0.05 was considered statistically significance. Statistical calculations were performed using SAS®9.4 (SAS Institute, Cary, NC).

## Results

87 patients were included in this cohort study. The average age was 67.4 ± 10.9 years old, and 77% were male. The study included roughly an equal percentage of ischemic vs. non-ischemic patients. Of the patients included, 26.4% were previously considered to have a high-risk upgrade. 48.3% had a failed CS lead implant, and another 25% had the CS lead turned off. At time of implantation ∼64% were demonstrating NYHA class III heart failure symptoms while 36% were demonstrating NYHA class II symptoms (Table 1). At time of implantation, 4 patients were in atrial fibrillation. Based on historical information 39/87 patients (44.8%) had a documented history of atrial arrhythmias.

**Table 1:** Patient demographic data.

Patient demographic data	Mean ± SD or *N* (%)
Age (years)	67.4 ± 10.9
Males	67 (77.0)
BMI (kg/m^2^)	30.6 ± 5.6
SBP (mmHg)	118.4 ± 17.5
DBP (mmHg)	70.0 ± 10.3
MAP (mmHg)	86 ± 11.2
Indication for implant	
High risk upgrade	23 (26.4)
Failed CS lead implant	42 (48.3)
CS lead turned Off	22 (25.3)
NYHA class at baseline	
Class II	31 (35.6)
Class III	56 (64.4)
Type of cardiomyopathy	
Ischemic	45 (51.7)
Non-ischemic	42 (48.3)

Representation of patient demographics of 87 patients included in study, reason for implant of WiSE-CRT system. Data presented as mean (± SD) or as percentage of the cohort. BMI, body mass index; SBP, systolic blood pressure; DBP, diastolic blood pressure; MAP, mean arterial pressure; NYHA, New York Heart Association.

Evaluation of echocardiographic parameters in this patient cohort comparing baseline values to 6 months post CRT implantation are found in Table 2. There was a statistically significant improvement in LV volumes, with a noted improvement in LVEF from 30.9 to 36.2 (*p* < 0.001) from baseline to 6 months.

**Table 2:** Echocardiographic evaluation pre and post CRT implantation.

Echo parameter	*N*	Baseline echo (mean ± SD)	*N*	6 month echo (mean ± SD)	*p* Value
LVEDV (mL)	87	208.9 (84.8)	87	182.6 (84.8)	0.0423
LVESV (mL)	87	148.1 (72.4)	87	122.1 (73.4)	0.0198
LV EF (%)	87	30.9 (8.04)	87	36.2 (9.88)	0.0001
LVGLS (%)	71	9.90 (3.66)	63	10.7 (3.42)	0.195
LVGCS (%)	72	11.0 (4.38)	64	12.6 (5.26)	0.0551
RVFAC (%)	85	39.9 (9.34)	71	39.2 (7.86)	0.617
TAPSE (cm)	86	1.73 (0.78)	81	1.72 (0.82)	0.936
Diastolic parameters					
E wave (cm/s)	86	87.0 (32.7)	86	78.7 (35.1)	0.111
A wave (cm/s)	63	78.7 (27.0)	66	77.9 (25.1)	0.862
E/A ratio	63	1.13 (0.65)	66	1.02 (0.59)	0.316
MVdct (ms)	86	189.6 (67.4)	85	206.3 (65.0)	0.101
TDI e’ septal (cm/s)	85	4.92 (1.74)	82	5.13 (1.79)	0.443
TDI e’ lateral (cm/s)	86	7.29 (2.97)	81	7.01 (2.63)	0.521
E/e’ ratio	85	15.9 (8.45)	81	14.2 (8.58)	0.200
LAVI (mL/m^2^)	87	39.5 (18.1)	83	38.7 (17.6)	0.771
RAP (mmHg)	78	6.01 (4.12)	78	5.88 (4.13)	0.844
Mitral regurgitation					
Vena contracta width (mm)	87	3.26 (2.54)	87	2.77 (2.55)	0.206

Evaluation of echocardiographic volumetric parameters, diastolic filling parameters, and mitral regurgitation vena contracta. Comparing baseline echocardiographic data to 6 months post implantation. LV, left ventricle; LVEDV, left ventricular end diastolic volume; LVESV, left ventricular end systolic volume; LVEF, left ventricular ejection fraction; LVGLS, left ventricular global longitudinal strain; LVGCS, left ventricular global circumferential strain; RVFAC, right ventricular fractional area change; TAPSE, tricuspid annular plane systolic excursion; MVdct, Mitral valve deceleration time; TDI, tissue doppler imaging; LAVI, left atrial volume index; RAP, right atrial pressure.

**Figure 1:**
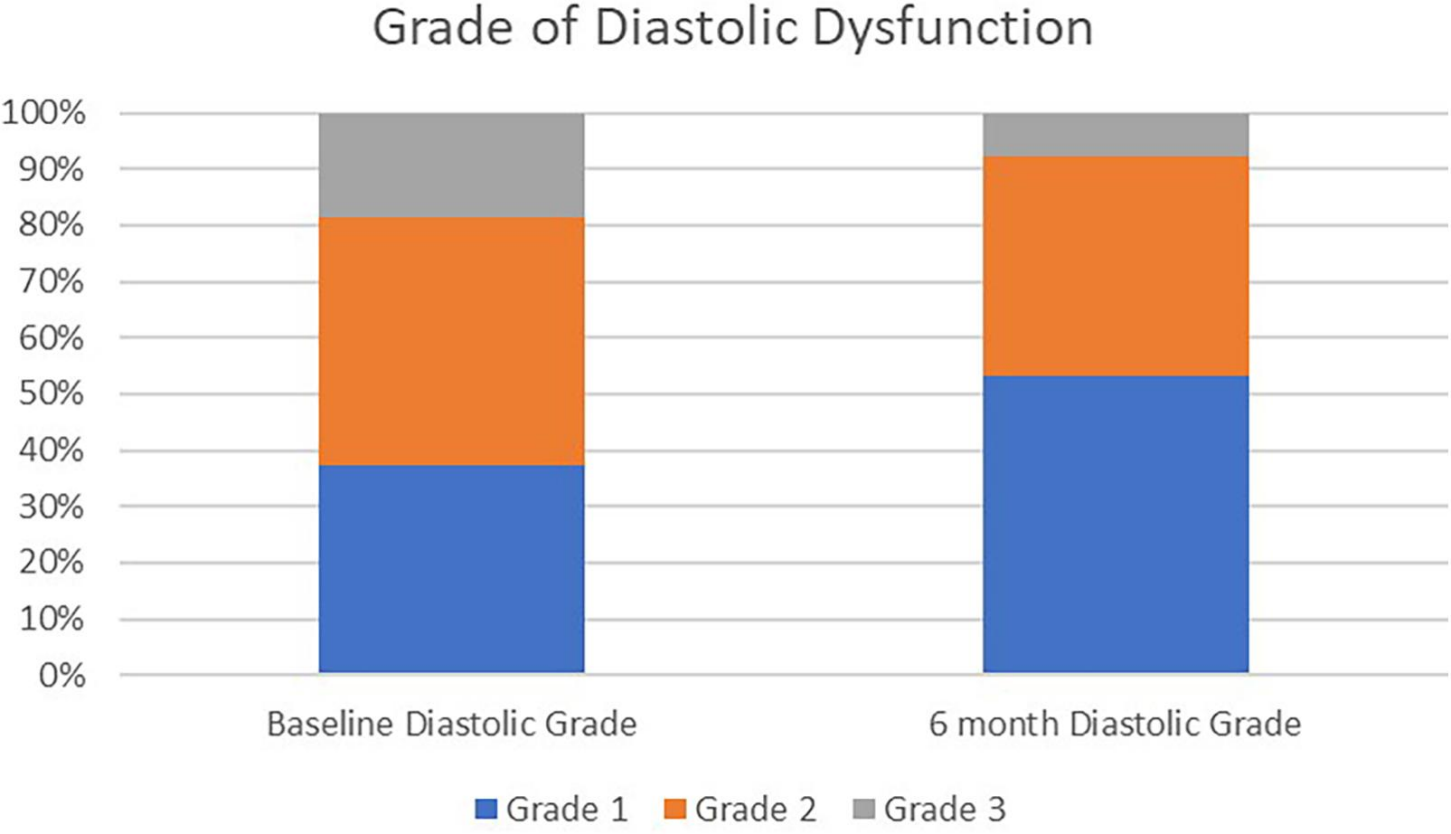
Grade of diastolic dysfunction from baseline to 6 months following WiSE-CRT implantation.

In the evaluation of individual diastolic parameters, there were several trends towards improved LV filling parameters and decreased left atrial filling pressures: decreased E wave, decreased E/e’ ratio, and increased mitral deceleration time (Table 2). Patients were categorized into grades of diastolic dysfunction based on current American Society of Echocardiography guidelines at both baseline and 6-months. The distribution of diastolic dysfunction showed significant improvement at 6-months follow-up (*p* < 0.001). There was a higher percentage of Grade I dysfunction (53.4% vs. 39.7% at baseline). This was associated with a reduction in Grade II (5.1% absolute decrease) and Grade III (8.6% absolute decrease) diastolic dysfunction. Patients who had indeterminate diastolic parameters at baseline and/or 6-months were not included in the evaluation of diastolic grading (Table 3 and Figure 1).

**Table 3:** Comparison of mitral regurgitation and diastolic grading.

Mitral regurgitation grade (0–4)	Baseline MR *N* (%)	6 month MR *N* (%)	*P* value
Grade 0	16 (18.4)	28 (32.2)	0.001
Grade 1	46 (52.9)	37 (42.5)	
Grade 2	21 (24.1)	21 (24.1)	
Grade 3	3 (3.5)	1 (1.2)	
Grade 4	1 (1.2)	0 (0.0)	
Diastolic dysfunction grade	Baseline diastolic grade *N* (%)	6 month diastolic grade *N* (%)	*P* value
Grade I	23 (39.7)	31 (53.4)	0.001
Grade II	26 (44.8)	23 (39.7)	
Grade III	9 (15.5)	4 (6.9)	

Evaluation of qualitative grading of degree of mitral regurgitation (*n* = 87) from baseline to 6 months. As well, evaluation of diastolic grade based on ASE criteria from baseline to 6 months (*n* = 58, matched cohort).

**Figure 2:**
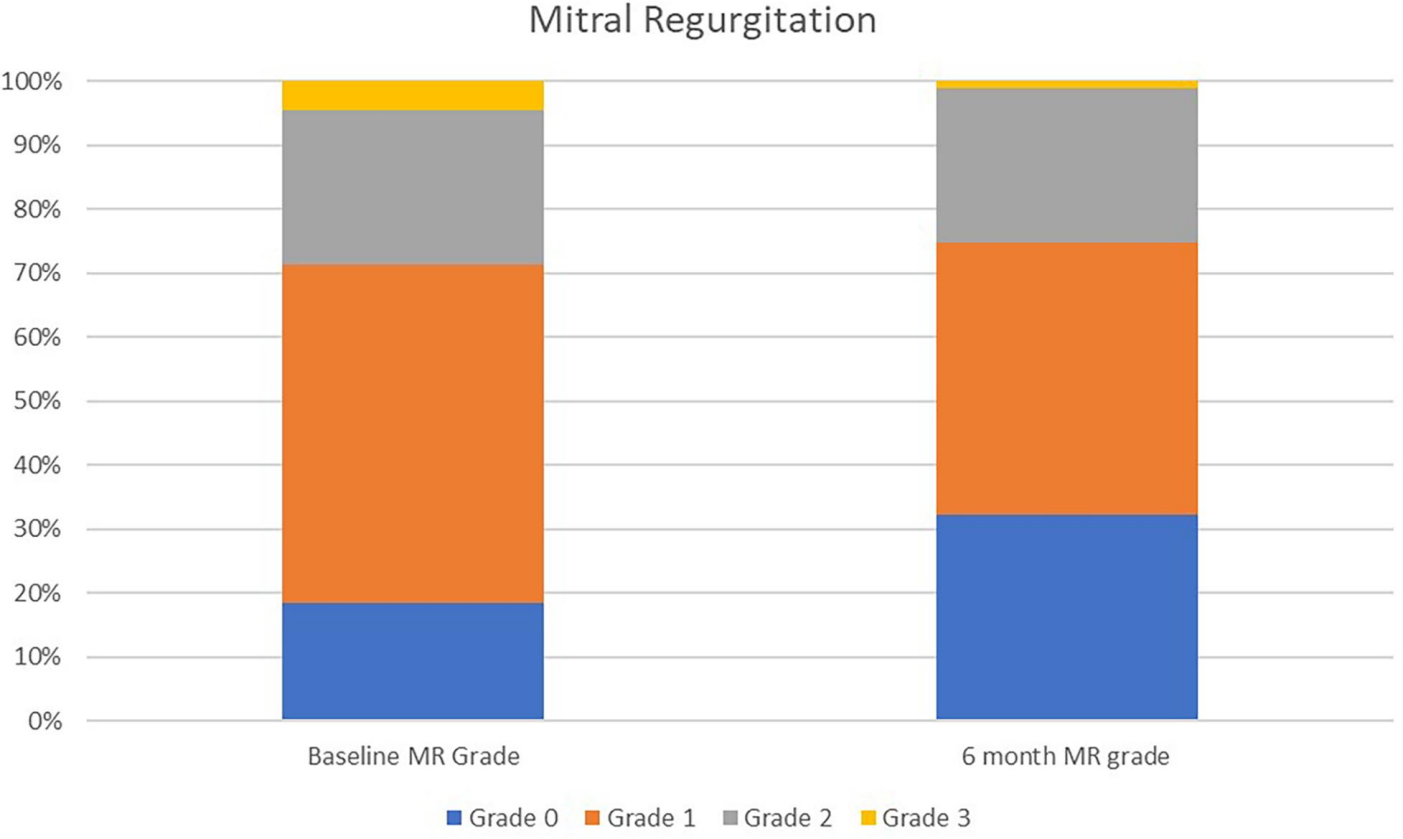
Grade of mitral regurgitation from baseline to 6 months following WiSE-CRT implantation. Grade 0 = no regurgitation; Grade 1 = mild regurgitation; Grade 2 = moderate regurgitation; Grade 3 = moderate to severe regurgitation.

Evaluation of MR demonstrated a slight decrease in the vena contracta width (3.26–2.77, *p* = 0.206). Grading metrics on a qualitative 0–4 scale did show a significant improvement in the grade of mitral regurgitation from baseline to 6 months (Table 3 and Figure 2).

A subgroup analysis was performed comparing patients with ischemic cardiomyopathy to those with non-ischemic cardiomyopathy (Table 4). Overall, comparing the delta change seen between baseline and 6 months between the two groups, there was only a statistically significant change noted in E/e’ ratio. In addition, the non-ischemic cardiomyopathy group had a significant reduction in E/e’ ratio from baseline to 6 months which was not observed in the ischemic cohort.

**Table 4:** Ischemic vs. Non-ischemic cardiomyopathy.

Echo parameter	*N*	Ischemic cohort delta change (6 month – baseline) (mean ± SD)	*N*	Non-ischemic CohortDelta change (6 month – baseline) (mean ± SD)	*p* Value
Diastolic parameters					
E wave (cm/s)	44	−8.29 (24.6)	41	−7.68 (25.2)	0.910
A wave (cm/s)	26	−3.77 (18.1)	28	−10.8 (19.4)	0.180
E/A ratio	26	−0.03 (0.43)	28	0.06 (0.37)	0.410
MVdct (ms)	43	9.53 (63.6)	41	21.3 (76.8)	0.450
TDI e’ septal (cm/s)	42	−0.05 (1.43)	39	0.46 (2.08)	0.200
TDI e’ lateral (cm/s)	41	−0.51 (3.23)	40	0.20 (2.43)	0.270
E/e’ ratio	41	0.18 (8.06)	39	−3.36 (6.31)	0.030
LAVI (mL/m^2^)	42	−1.76 (20.3)	41	−1.80 (21.6)	0.990
Mitral regurgitation					
Vena contracta width (mm)	45	−0.51 (1.59)	42	−0.48 (1.92)	0.940

Subgroup analysis comparing diastolic and mitral regurgitation parameters in patients with ischemic cardiomyopathy vs. nonischemic cardiomyopathy. Data presented as the delta change in each group from baseline to 6 months. MVdct, mitral valve deceleration time; TDI, tissue doppler imaging; LAVI, left atrial volume index.

## Discussion

This study evaluated echocardiographic parameters in patients with the WiSE-CRT system beyond those associated with LV remodeling that were reported in the primary analysis of the SOLVE-CRT trial The findings in our study demonstrated significant improvements in summative diastolic grading, with trends toward improvement in individual diastolic measurement., It also showed an improvement in the degree of MR 6-months after device implantation based on qualitative assessment.

The present study showed improvements in LV remodeling along with improvements in LV filling parameters and diastolic grading. The cohort of patients in this study showed a significant improvement in LVEF from 30.9% to 36.2% (*p* < 0.001) from baseline to 6 months. In addition, multiple parameters representative of LV filling pressures improved over the 6-month time period. We observed a reduction in the mitral E and A waves which is suggestive of lower left atrial filling pressures. Although not statistically significant, there was a noted trend towards a reduction in E/e’ ratio from 15.9 to 14.2 (*p* = 0.20). The E/e’ ratio on echocardiogram becomes representative of the pulmonary capillary wedge pressure based on Nagueh's principle (10). The reduction in this parameter indicates an improvement in overall volumes and intra-cardiac pressures. Previous small studies have shown having a reduction in E/e’ ratio leads to improved outcomes and reduced risk of heart failure hospitalizations and cardiac death (12–14). The reduction in E/e’ was more noticeable in the non-ischemic cohort of patients in which a statistically significant reduction was noted. When applying the American Society of Echocardiography diastolic grading classifications to this patient cohort, we observed an overall improvement in diastolic grading. The majority of patients 6 months after implantation were demonstrating a grade I filling pattern, with reductions from baseline in those with grade II and II filling patterns. This is congruent with the reduction noted in individual LV filling parameters and portrays the overall trend towards a reduction in filling pressures and improvement in LV filling dynamics.

The findings in our study are similar to previous studies evaluating diastolic filling parameters in patients undergoing transvenous CRT devices. Two previous studies, Doltra et al. (3) and Waggoner et al. (8), noted a change in diastolic parameters was related to positive changes in LV volumes and systolic function following CRT insertion. In Doltra et al. (3), 250 patients were evaluated following transvenous CRT placement. 54% of these patients demonstrated reverse remodeling of the LV with improvements in LV volumes and overall systolic function. In these patients with reverse remodeling, they were noted as well to have improvements in diastolic parameters and diastolic grading at 1 year. In those without a positive remodeling response, these improvements in diastolic parameters were not noted, and in fact there was an increase in E/e’ ratios. A study by Waggoner et al. (8); showed similar findings in which those with evidence of reverse remodeling showed improvements in diastolic parameters.

Our study included all patients in the analysis and did not evaluate patients based on the remodeling response to CRT pacing. Based on prior studies, the inclusion of responders and non-responders may have reduced the impact on diastolic parameters, particularly given the small sample size. However, even with inclusion of all patients, our data still demonstrated a trend towards improvement in diastolic parameters with non-significant changes in E waves, E/A ratio, E/e’ ratio, and mitral valve deceleration time. The improvement noted in diastolic grading seen in our study is similar to that observed in Doltra et al. (3).

The second key finding of this study is the improvement in functional MR, which is commonly associated with ischemic and non-ischemic cardiomyopathies. Previous studies have demonstrated an improvement in MR after CRT is linked to an improvement in survival (15, 16). Traditional transvenous CRT pacing has led to an improvement in overall MR severity (17, 18). Our data evaluating the WiSE-CRT left endocardial pacing system revealed an improvement in MR similar to that seen with traditional CRT. At 6 months, qualitative MR grading showed a significant improvement. Approximately 75% of patients in the cohort were classified as having either Grade 0 or 1 mitral regurgitation on their follow-up echocardiogram. Additionally, we saw a trend towards a reduction in vena-contracta jet width from baseline to 6 months.

## Conclusions

The WiSE-CRT system is an innovative approach to biventricular pacing for patients who had previously been unable to achieve successful CRT therapy. In addition to the previously published improvements in LV volumes and LVEF, this study demonstrated a positive impact on diastolic filling parameters and qualitative MR. The improvement seen in diastolic grading and trend towards improvement in individual diastolic parameters may have a positive correlation with improvements in clinical symptoms and patient outcomes. Our data also supports that this novel technique for CRT correlates with an improvement in the severity of MR as seen with traditional BiVP. This study adds to the current literature and supports the benefits associated with the WiSE-CRT pacing system for previously untreatable or high-risk patients.

## Study limitations

The small cohort of patients and the retrospective nature of our analysis represent major limitations of the data. This was a secondary analysis of a trial designed to evaluate the impact of a novel synchronization method on LV remodeling. Therefore, the echocardiographic protocol did not include all parameters desirable for a complete assessment of diastolic and valvular pathology. 19 of the 87 patients in this study were assigned indeterminate diastolic function. Additionally, although we saw an improvement in summative diastolic grading from baseline to 6 months, there was no statistically significant difference in the evaluation of individual diastolic parameters. We suspect that this is related to the small sample size in this cohort. There was a trend towards improvement in multiple diastolic parameters. It is plausible that with a larger patient cohort or extended follow-up interval, individual parameters may show more significant improvement, but that cannot be supported by this data.

Regarding the evaluation of MR, the initial study's imaging protocol was not designed for quantitative assessment of mitral regurgitation. Given this we were unable to utilize PISA evaluation to quantify MR severity. While we acknowledge our reliance on qualitative MR assessment, this was performed in the context of an echocardiographic core lab with extensive experience in analysis and a very limited number of reviewers (*n* = 3) which we feel reduces the risk of subjective variability.

## Data Availability

The raw data supporting the conclusions of this article will be made available by the authors, without undue reservation.
